# Delayed Treatment for People Living with HIV in China, 2004–2016: An Analysis of An Observational Cohort

**DOI:** 10.3390/ijerph17051809

**Published:** 2020-03-10

**Authors:** Junfang Xu, Anders Sönnerborg, Liangmin Gao, Peicheng Wang, Jennifer Z.H. Bouey, Feng Cheng

**Affiliations:** 1 Center for Health Policy Studies, School of Public Health, Zhejiang University School of Medicine, Hangzhou 310058, China; xujf2019@zju.edu.cn; 2 Divisions of Infectious Diseases and Clinical Microbiology, Karolinska Institutet, 17177 Stockholm, Sweden; Anders.Sonnerborg@ki.se; 3 Research Center for Public Health, School of Medicine, Tsinghua University, Beijing 100084, China; glmdhx@126.com (L.G.); yingziwilk@163.com (P.W.); 4 Institute for International and Area Studies, Tsinghua University, Beijing 100084, China; 5 Department of International Health, School of Nursing & Health Studies, Georgetown University, Washington, DC 20037, USA; Jennifer.bouey@georgetown.edu

**Keywords:** delayed treatment, people living with HIV, observational cohort

## Abstract

Early universal access to antiretroviral treatment (ART) is critical in the control of the HIV epidemic. However, prompt initiation of ART remains problematic in China. This study analyzed the late testing and lag time between HIV diagnosis and initiation of ART from 2004 to 2016 and identified the risk factors for delayed initiation of ART. Data from 16,957 people living with HIV were abstracted from a hospital electronic health record database and a case report database for AIDS prevention and control in Yunnan province. Reasons for delayed initiation of ART were categorized into late testing, defined as CD4 count of < 350 cells/μL at baseline HIV diagnosis, and delayed access, defined as a lag time of > 1 month between the diagnosis and initiation of ART. Binary logistic regression models were used to identify risk factors for late testing and delayed access. The CD4 counts at diagnosis increased from 201 ± 147 cells/μL (mean ± SD) in 2004 to 324 ± 238 cells/μL in 2016 (*p* = 0.024). The CD4 count was higher for persons < 45 years, unmarried, and men who have sex with men (MSM) (356, 357, and 409 cells/μL, respectively) compared to their peers in 2016 (*p* < 0.05). The lag time from diagnosis to initiation of ART was significantly reduced from 59.2 months in 2004 to 0.9 months in 2016 (*p* < 0.05). The shorter lag time over the years was consistent when analysis was stratified by sex, age, marital status, and transmission routes, even though the lag time for people using drugs was longest in 2016 (> 2 months versus 0.82 and 0.72 month of heterosexuals and MSM, respectively). Compared to their peers, married persons (AOR = 0.63, 95%CI: 0.57, 0.69) were less likely to have delayed access to ART, and drugs-using patients (AOR = 3.58, 95%CI: 2.95,4.33) were more likely to have delayed access to ART. Late testing rather than delayed access to ART after a diagnosis remains problematic in China, although improvements have been seen for both parameters from 2004 to 2016. Our data highlight the importance of continued efforts to promote early diagnosis of HIV to prevent transmission, morbidity, and early mortality in HIV infection.

## 1. Introduction

By the end of 2018, 37.9 million people were living with HIV (PLHIV) globally and 1.7 million new HIV cases were diagnosed that year [1]. Declines in the rates of new HIV infections among adults have however slowed, but certain regions in the world continue to experience increases in new diagnoses [2]. In China, 958,000 people were living with HIV/AIDS [3,4], with a prevalence of 0.05% nationwide but > 1% in some specific counties (e.g., Hekou, Liangshan) [5]. From January to October 2019, 131,000 new HIV infections were found [5]. In addition, around 3000 new diagnoses among 15- to 24-year-olds were found each year, mostly (82.4%) among men who have sex with men (MSM) [3]. The new incidence among adults of > 60 years rose from 9167 in 2011 to 21,102 in 2016, of whom most (94.2%) were infected heterosexually [5]. Thus, the HIV epidemic in China is continuing while the demographic patterns are changing.

To date, antiretroviral treatment (ART) is very effective in reducing mortality among PLHIV, and achieving undetectable HIV-RNA can prevent HIV transmission [4,5]. Moreover, if ART is initiated early after diagnosis, regardless of CD4+T-cell count, HIV transmission in the population may be reduced even further [6]. Therefore, early diagnosis, linkage to, and retention in care with ART initiation in infected individuals has for many years been a priority of AIDS prevention and control programs worldwide [7,8,9].

From the turn of the century, the Chinese government has adjusted the thresholds for initiating ART to promote early treatment (Table 1). To achieve the 90–90–90 goal announced by the Joint United Nations Programme on HIV and AIDS [10], Chinese center for disease control and prevention (China CDC) recommended treatment guidelines that ART should be initiated in all PLHIV, regardless of CD4 level in 2016 (Table 1). However, many PLHIV still present late for diagnosis and initiation of ART [11]. A national cohort study in China with 123,605 HIV/AIDS patients has revealed that beginning ART within 30 days after HIV/AIDS diagnosis was associated with significantly reduced treatment drop-out and virologic failure [12]. Therefore, ensuring timely access to ART for PLHIV is beneficial for both individuals and public health.

To address if there have been any improvements with regard to early diagnosis and early initiation of ART since free ART was first offered in China in 2003 and also to provide evidence to inform interventions to promote early ART, we analyzed the CD4 cell counts at diagnosis and the lag time between diagnosis and initiation of ART among PLHIV from 2004 to 2016 in China and identified factors associated with delayed initiation of ART.

## 2. Methods

### 2.1. Data Sources and Study Samples

Data were abstracted from a hospital electronic health record database (EHR) and a case report surveillance database for AIDS prevention and control in Yunnan province in southwest China. The province has the highest prevalence of HIV in China, with 111,700 diagnosed PLHIV by October 2019, accounting for 14% of all PLHIV in China [18,19]. The ART program in Yunnan is consistent with national HIV/AIDS-related policies.

The databases routinely record patients’ socio-demographics (e.g., gender, age, marital status, and place of residence), clinical information (e.g., time of diagnosis, CD4 cell count, and viral load), and ART-related information (e.g., time of initial ART, regimen, and adverse effects). A unique personal index (i.e., therapeutic number) was used to consolidate the two databases and remove duplicates. Patients younger than 18 years were excluded. This resulted in a sample of 16,957 PLHIV, including dead patients. Confidentiality was protected as part of the management of individual information and the processing of personal data. The study protocol and consent procedure were approved by the Institutional Review Board for Human Subject Research at the Research Centre for Public Health at Tsinghua University (Code: 20163000254).

### 2.2. Measurements

The reasons of delayed initiation of ART were categorized into: (i) late testing: patients with CD4 count < 350 cells/μL at baseline HIV diagnosis [20]; and (ii) delayed access: a lag time of > 30 days after HIV diagnosis to initiation of ART, a definition used in the recent national cohort study in China [12]. The lag time was expressed as the number of months.

### 2.3. Data Analyses

Mean, SD, and percentages were used to describe the demographic characteristics of study population, lag time, and CD4 counts. Multiple regression models (i.e., binary logistic regression model) were used to identify risk factors associated with delayed ART, including late testing and delayed access to ART. The following data were included as independent variables: gender, age, marital status, ethnicity, education level, employment, mode of transmission, year of diagnosis, and CD4 count at diagnosis. In addition, liner regression was used to examine whether the lag time and CD4 counts statistically decreased in the last 13 years. All statistical analyses were based on a pooled dataset from the two databases. SAS 9.2 software (SAS Institute Inc., Cary, North Carolina, USA) was used with an alpha level of 0.05.

## 3. Results

A total of 16,957 PLHIV receiving initial ART between 2004 and 2016 were included (Table 2). The majority (71.6%) were male; mean age of 40 years (SD: 13.34); half (55.4%) were married; and more than one half (56.8%) were infected through heterosexual transmission, 32.5% through intravenous drug use, and 4.4% through MSM transmission.

**Figure 1:**
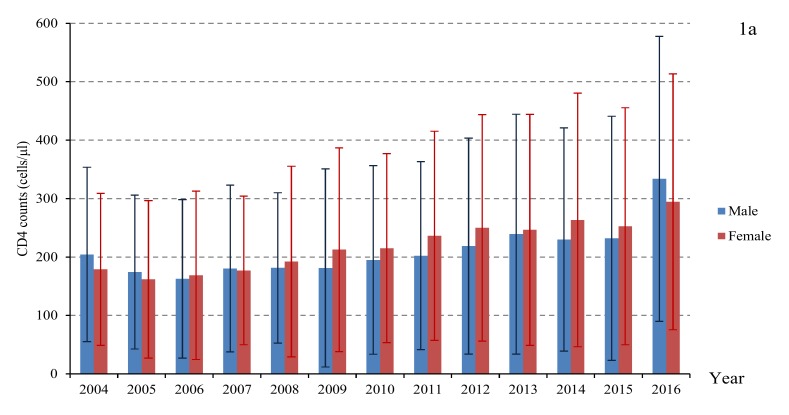

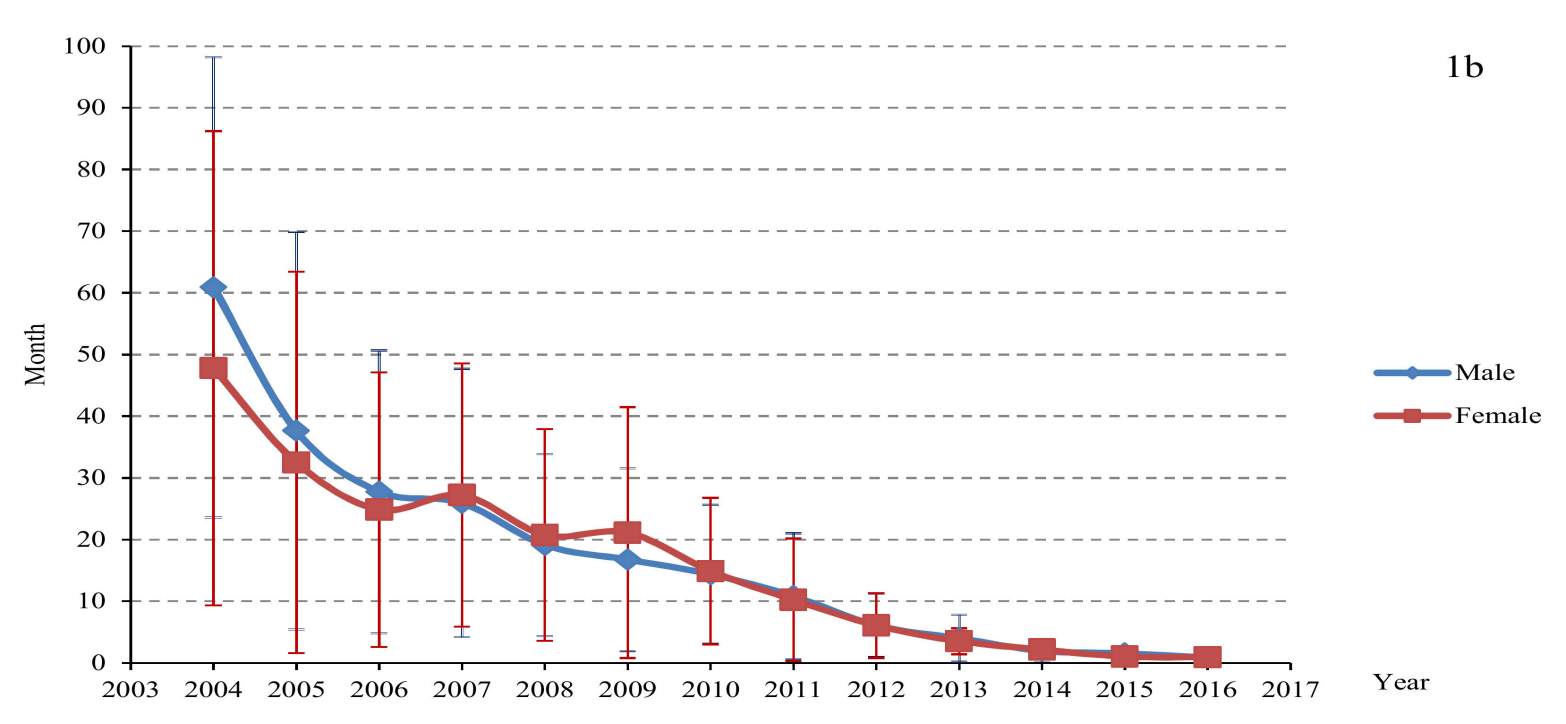
The CD4 counts at diagnosis (**a**) and lag time between HIV diagnosis and initial ART (**b**) by gender.

**Figure 2:**
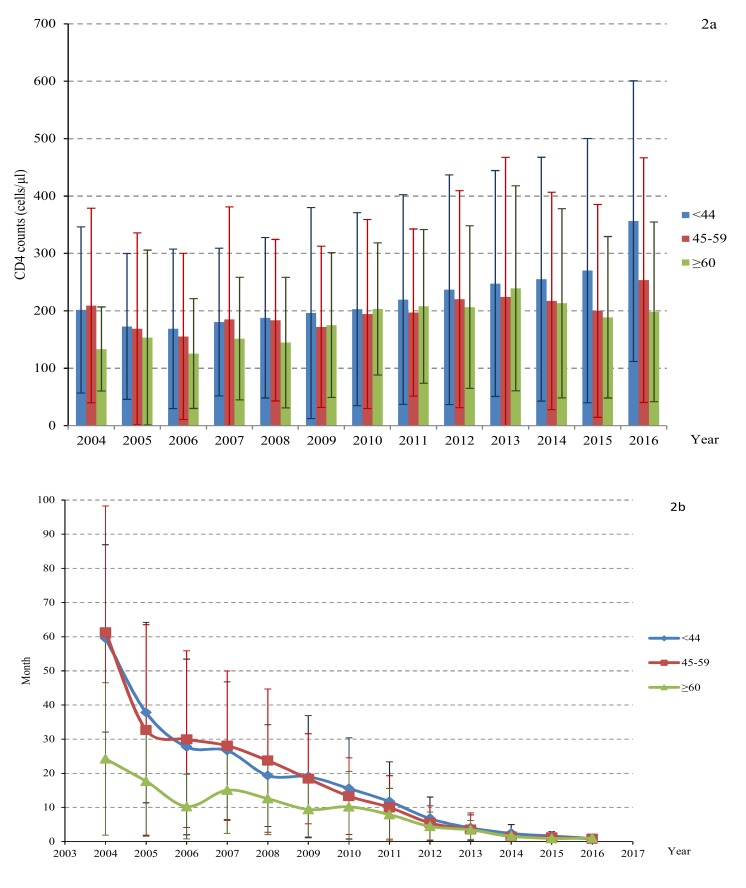
The CD4 counts at diagnosis (**a**) and lag time between HIV diagnosis and initial ART (**b**) by age.

**Figure 3:**
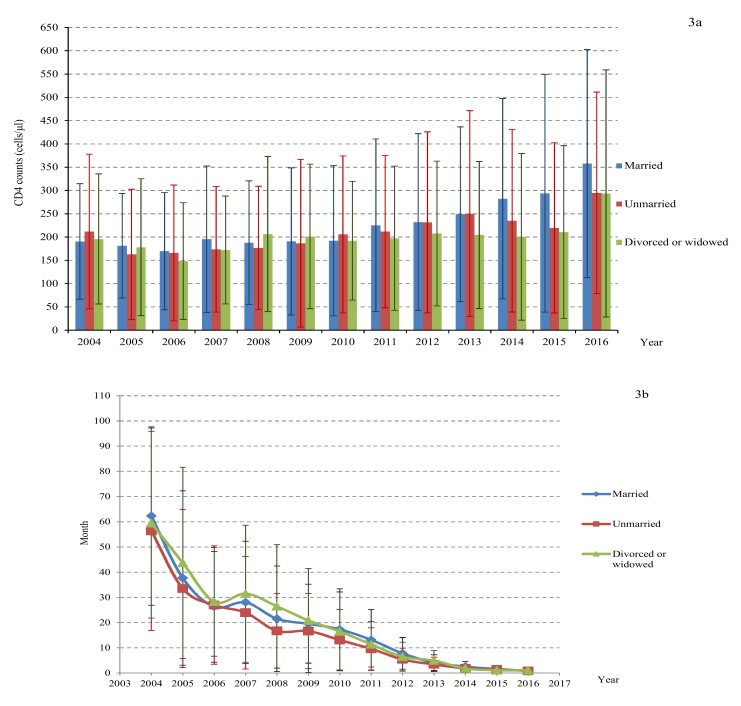
The diagnosed CD4 counts (**a**) and lag time between HIV diagnosis initial ART (**b**) by marital status.

**Figure 4:**
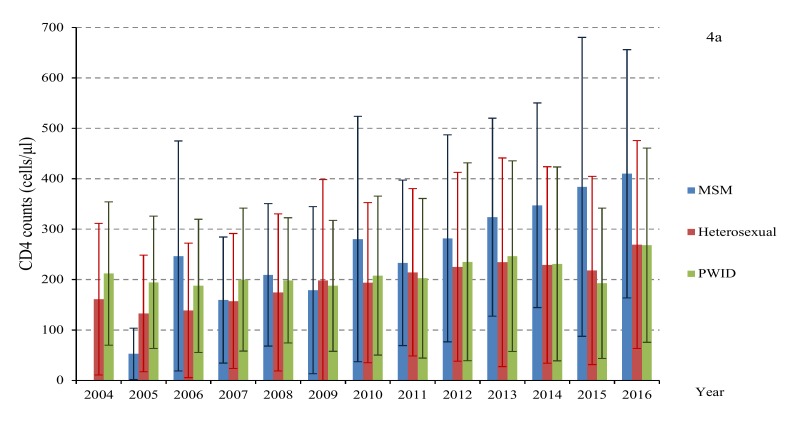

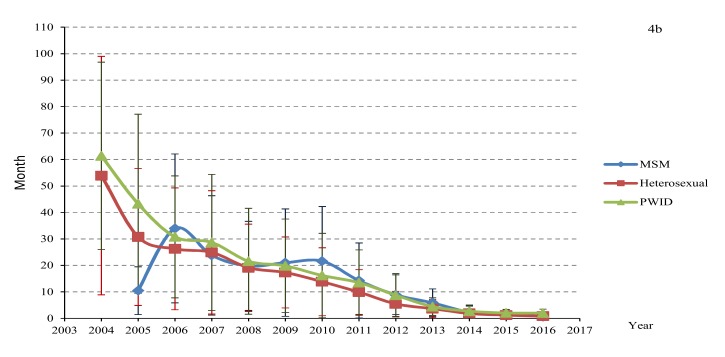
The CD4 counts at diagnosis (**a**) and lag time between HIV diagnosis and initial ART (**b**) by transmission routes.

The CD4 counts at diagnosis of the total study population increased from 201 ± 147 cells/μL in 2004 to 324 ± 238 cells/μL in 2016(*p* = 0.024) (Table 3). At subanalysis (Figure 1, Figure 2, Figure 3 and Figure 4), the CD4 counts also showed a slight increase for all groups (e.g., male and female, different age, married and single, persons with intravenous drug use (PWID) and MSM) from 2004 to 2016. Generally, the CD4 count was higher for unmarried people, below 45 years old and MSM, which increased to 358 cells/μL, 356 cells/μL, 410 cells/μL in 2016, respectively (*p* < 0.05).

The average lag time between HIV diagnosis and initiation of ART was reduced significantly from 59.2 months in 2004 to 0.9 months in 2016 (*p* < 0.05). The trend analysis of the lag time by different genders, ages, marital status, and transmission routes showed a significant decline over time (Figure 1, Figure 2, Figure 3 and Figure 4). However, the lag time for PWID was still more than 1 month (2.0 months) in 2016.

Univariate logistic regression showed that married and heterosexual patients were more likely to be diagnosed late, while there were no statistical differences compared with their peers in multiple regression analysis (AOR: 1.23, 95%CI: 0.79,1.91 and AOR: 1.30, 95% CI: 0.79,2.14 respectively) in Table 4. In contrast, PWID were more likely than MSM to experience delayed access to ART (AOR: 3.58, 95% CI: 2.95, 4.33).

## 4. Discussion

The present study of delay to antiretroviral therapy (ART) focuses on the HIV testing and initiation of ART during last 13 years in China. We found that time to ART initiation in Yunnan province has improved from 2004 to 2016 with regard to both parameters, although major challenges still persist especially with regard to late diagnosis.

Late diagnosis is a common problem all over the world and few countries reach the UNAIDS goal that 90% of HIV infected individuals in a country should be identified [10]. The reasons are multifactorial, including both patients delay and doctors delay of testing [21]. This problem persists globally and in China despite the wide availability of ART. In fact, considering the adjustment of threshold by National Health Commission of the People’s Republic of China for initiating ART from CD4 < 200 cells/μL to CD4 > 500 cells/μL, we hypothesized that the initial CD4 counts when starting ART should have increased markedly in past 13 years. Obviously, this hypothesis was disproven by the data. In contrast, the low CD4 counts at diagnosis between 2004 and 2016 imply that the HIV infections were still tested late in China. The potential consequences are severe because only persons who initiate treatment at higher CD4 levels experience lower morbidity and mortality than those who delay testing [22,23]. Moreover, PLHIV who are unaware of their HIV status are less likely to take steps to prevent onward transmission to others [24,25]. Therefore, more efficient programs for early testing of HIV are urgently required. In our study, compared with heterosexual people, the CD4 counts at diagnosis of MSM were higher and increased further to 409 cells/μL in 2016. This is likely to be a consequence of intensified HIV testing strategies, such as HIV testing at STI clinics and the encouragement of high-risk MSM to test for HIV every 3 months [26]. In addition, internet facilities (i.e., web-based platforms, such as dedicated websites featuring online risk assessment and appointment making and crowd-sourced service promotion messages and dissemination via participants’ microblog accounts and social media profiles) for HIV testing and partner notification for MSM have also been established [27,28]. It should be noted that the increased prevalence of HIV among MSM in recent years is driven by college students aged 18–24 years old [29]. In contrast for heterosexuals, effective innovative HIV testing strategies are lacking, which results in late testing with a low CD4 count, a situation also found in some other countries [30].

In the study, a major achievement was that the lag time was significantly reduced from 2004 to 2016 for all groups living with HIV/AIDS. The attention given to this population by the central government has most likely contributed to this important change. Such attention includes a series of measures, for example, the education on awareness, perception of HIV risk, stigma of HIV/AIDS disease, “Four Frees and One Care” policy (which provides free ART for all AIDS patients in financial difficulty; free schooling for AIDS orphans and children of AIDS patients; free counseling and prevention measures to prevent mother-to-child-transmission for HIV-infected pregnant women; free HIV antibody testing and counseling; and care to AIDS patients and their families), as well as improvements in HIV care [31,32]. However, delayed entry to ART of PWID, whose lag time was more than 2 months in 2016, still remains problematic, an experience also found in other countries [33,34]. This result is not unexpected because PWID are difficult to enroll and retain in care, although they receive HIV tests in drug rehabilitation centres [26].

The factors associated with delayed entry to ART differed from those associated with late testing. Married people tended to enter ART early, but they did not show early testing. It may be that AIDS interventions are more difficult to reach married patients, who are not considered as a high-risk population [30]. Moreover, married people may be afraid of the impact (e.g., stigma) of potential positive results on their families and therefore may delay testing. In addition, unmarried people tend to delay initial ART compared with married people. This may be because these young, single, and currently healthy people are not aware the importance of early ART since the body does not show any symptoms of disease [35].

### 4.1. Implications for Health Policy

In China, although ART is available and free for all PLHIV, delayed ART especially due to delayed testing is still a major public health problem. This can only be improved if people at risk test for HIV earlier. Therefore, early testing and entry into ART are crucial to optimize treatment outcomes of PLHIV and to prevent further spread of HIV. One method for identifying PLHIV at an earlier stage is to broaden HIV screening practices from passive (hospital based screening driven by patient initiation and symptoms) to active screening (e.g., voluntary, routine, and more frequent HIV screening of the high risk population). It is known that CD4 counts of HIV patients detected at blood donation, premarital examination, and physical examination of entry-exit personnel actively are higher than in patients identified by passive detection [36]. Moreover, a combination of both community-based and general practitioner initiated interventions for HIV testing, removal of stigma, as well as working towards acceptance of verbal informed consent for testing are important. Such interventions will be especially important for the attainment of the UNAIDS 90–90–90 targets in China. In addition, interventions to improve linkage to ART should be implemented alongside HIV screening strategies, particularly in marginalized groups such as PWID, who tend to be diagnosed early but are given ART late. Effective methods could be to make HIV testing and ART a one-stop service in medical institutions or explore follow-up and treatment referral services. Furthermore, interventions on the adherence of ART and risk behaviors reduction for HIV positive people are also important to avoid further onward transmission.

### 4.2. Strengths and Limitations

One of the strengths of our study is the large number of patients included from a nonselective province wide, longitudinal cohort, which made it possible to study the time trends over a decade period. This study also has limitations. First, due to the retrospective nature of the study design, only available clinical/demographic parameters were analyzed. In addition, considering a large number of missing data on some variables (e.g., employment, ethnicity, and education level), the related results may not represent the actual demographic characteristics of PLHIV. Additionally, since we did not have any data on lost-to-follow up after diagnosis, both the CD4 cell count at initiation of ART and the lag time to initiation of ART may be skewed in our study. An additional potential bias is that the CD4 cell counts at diagnosis may be falsely low since patients with primary HIV infection (PHI) may have been included. Such patients are known to have low CD4 cells during PHI which thereafter increase. Second, although there are a significant number of people living with HIV/AIDS in Yunnan, our data may not be representative of the entire people living with HIV considering the great variety of geographic and cultural backgrounds in China. Further prospective studies with larger sample sizes from other regions are needed to elucidate this issue in China. Third, considering that patient data from all years were combined in the models, risk factors for delayed treatment initiation may change over time.

## 5. Conclusions

In conclusion, the lag time between HIV diagnosis and initiation of ART was reduced significantly from 2004 to 2016. However, CD4 counts at diagnosis did not markedly increase despite the fact that the threshold for initiating ART increased from 2003 to 2016. Therefore, late testing, rather than delayed entry to ART after diagnosis remains problematic. This reveals a need to develop interventions (e.g., broaden HIV screening practices from passive to active screening in general population, build a model of routine, voluntary, and more frequent HIV screening in high risk population) that increase HIV testing and facilitate earlier entry into care, with special priority for married people.

## Figures and Tables

**Table 1:** The thresholds for initiating antiretroviral therapy over time in Chinese national treatment guidelines.

Year	Treatment Thresholds
2002 [13]	CD4 <200 cells/μL
2008 [14]	CD4 <350 cells/μL
2014 [15]	CD4 <500 cells/μL
2016 [16,17]	No threshold. All are recommended treatment.

**Table 2:** Characteristics of people infected with HIV during 2004–2016.

Variables	Items	Value	Percentage (%)
Gender	Male	12,144	71.6
	Female	4813	28.4
Age (years, mean ± SD)	All	40.0 ± 13.3	--
Marital status	Unmarried	4598	27.1
	Married	9390	55.4
	Divorced/Widowed	2886	17.0
	Missing	83	0.5
Employment	Worker	719	4.2
	Farmers	4129	24.4
	No work/retired	596	3.5
	Others	898	5.3
	Missing	10,615	67.9
Ethnicity	Han	4664	27.5
	Others	1710	10.1
	Missing	10,583	72.5
Education level	Primary school and below	2157	12.7
	Middle school	2535	14.9
	High school	857	5.1
	University and above	708	4.2
	Missing	10,700	63.1
Mode of transmission	Homosexual	751	4.4
	Heterosexual	9629	56.8
	Intravenous drug use	5508	32.5
	Others	997	5.9
	Missing	72	0.4
Year of ART initiation *	2004	841	4.9
	2005	838	4.9
	2006	1061	6.3
	2007	1362	8.0
	2008	1590	9.4
	2009	1510	8.9
	2010	1354	8.0
	2011	1562	9.2
	2012	1606	9.5
	2013	1556	9.2
	2014	1572	9.3
	2015	1229	7.3
	2016	816	4.8
	Missing	60	0.4

* The value is the number of HIV positive people receiving ART initially by year of ART initiation.

**Table 3:** CD4 counts at diagnosis and lag time between HIV diagnosis and initiation of antiretroviral therapy.

Year	Number *	CD4 Counts (Cells/μL) *	Lag Time (Months)
2004	841	201 ± 147	59.2 ± 37.7
2005	838	172 ± 132	36.6 ± 36.6
2006	1061	164 ± 138	27.0 ± 32.7
2007	1362	179 ± 139	26.3 ± 30.2
2008	1590	184 ± 139	19.6 ± 25.4
2009	1510	190 ± 171	18.0 ± 22.6
2010	1354	201 ± 162	14.5 ± 18.1
2011	1562	212 ± 167	10.7 ± 14.1
2012	1606	228 ± 188	6.1 ± 9.2
2013	1556	241 ± 203	3.9 ± 6.7
2014	1572	239 ± 199	2.0 ± 4.0
2015	1229	238 ± 207	1.4 ± 2.8
2016	816	324 ± 238	0.9 ± 1.6

Note. mean ± SD. Patients whose initial ART years were missing were excluded from the analysis. * The value is the number of HIV positive people receiving ART initially by year of ART initiation.

**Table 4:** Multiple regression analysis of late testing and delayed access to antiretroviral therapy (ART).

Variables	Late Testing	Delayed Access to ART
Covariate ^a^	AOR (95%CI)	AOR (95%CI)
Gender		
Female	1.15 (0.84,1.56)	0.88 (0.81,0.97)
Male (Ref)	1	1
Age (continuous)	0.69 (0.54,0.87)	0.69 (0.65,0.72)
Marital status (*p* < *0*.001)		
Married	1.23 (0.79,1.91)	0.63 (0.57,0.69)
Divorced or widowed	0.99 (0.68,1.46)	0.75 (0.66,0.85)
Unmarried (Ref)	1	1
Ethnicity		
Others	1.22 (0.93,1.61)	0.912(0.71,1.17)
Han (Ref)	1	1
Education level (continuous)	1.04 (0.90, 1.20)	0.88(0.80, 0.97)
Employment (*p* < *0*.001)		
Farmers	0.74(0.52,1.06)	1.03(0.77,1.38)
No work or retired	0.89(0.62,1.28)	1.24(0.92,1.67)
Others	0.69(0.48,1.00)	1.30(0.93,1.82)
Worker (Ref)	1	1
Mode of transmission (*p* < *0*.001)**		
Heterosexual/MSM (Ref)	1.30(0.79,2.14)	0.96(0.80,1.14)
PWID/ MSM (Ref)	1.16(0.74,1.82)	3.58(2.95,4.33)
PWID/ Heterosexual(Ref)	0.89(0.50,1.60)	1.04(0.84,1.30)
Year of diagnosis (continuous)	1.13(1.09,1.18)	1.06(1.03,1.08)
CD4 count at diagnosis(Continuous)	-	1.27(1.21,1.33)

Note. AOR = adjusted odds ratio; CI = confidence interval. ^a^ For variables with more than 2 categorical response options, we calculated the global *p* values. Global *p* values suggest evidence of significance for marital status (*p* < *0*.001), employment (*p* < *0*.001), and mode of transmission (*p* < *0*.001) on delayed entry to ART and late testing. ** MSM: men who have sex with men: PWID: persons with intravenous drug use. Each AOR means the odds ratio after incorporating other factors.
